# Non-Panel Men and the "Medical Directory"

**Published:** 1914-04-25

**Authors:** Arthur J. Brock

**Affiliations:** Edinburgh


### THE EDITOR'S LETTER-BOX.

### Non-Panel Men and the "Medical Directory."

*To the Editor of* The Hospital.

Sir,—Dr. F. L. Nicholls suggests that non-panel men are "entitled to some distinction" (such as special mention in the *Medical Directory*), because "they have in many instances suffered financially, if not mentally, by the strain," etc. He possibly forgets that also in many instances they have not so suffered, and that therefore the reward would in many instances be bestowed on people who did not deserve it. Further, the fact that this "distinction" was not bestowed on panel men might seem to suggest that panel men (as such) did not deserve distinction.

The fact, of course, is that there are black and white sheep in both flocks. We are all perfectly aware that many men, though disliking the panel, have been forced on to it from economic pressure. These men deserve our sympathy, not our censure. Others appear to like the panel. Why, then, should they not be allowed to stay on it?

No man can do good work unless he likes his job. *De gustibus non est disputandum.* If a free medical service is really better—*i.e.*, more effective—than one run by officials, surely it may be left to time to prove it so.

Lastly, we cannot eat our cake and have it too. There is a strange impression abroad at present that we may get liberty without paying for it. A bigger mistake was never made. In these days of democracy—*i.e.*, rule by the barristers—the price of freedom, like the rates, is going to go steadily up, and the sooner we reconcile ourselves to the fact the better. If one has expensive tastes, it is futile to grumble when the bill comes in.—I am, etc.,

Edinburgh, April 17, 1914. Arthur J. Brock, M.D.

